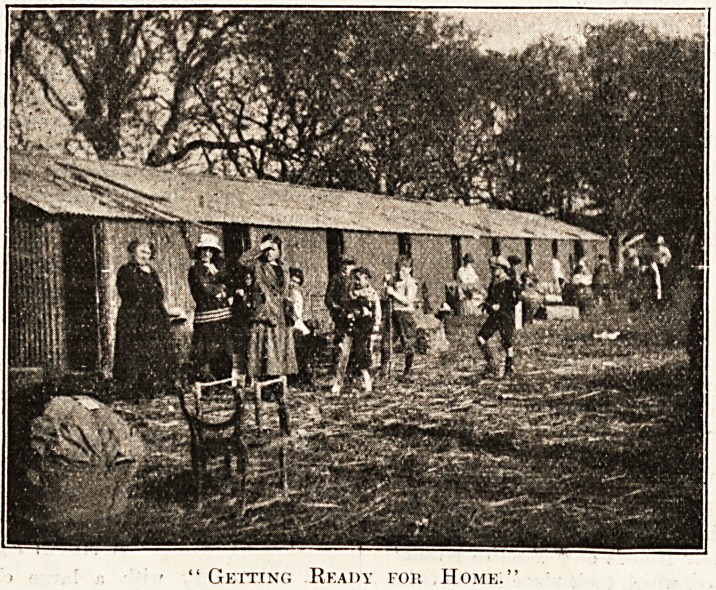# The General and Medical Work of a Deserving Mission

**Published:** 1921-01-22

**Authors:** 


### AMONG THE HOP-PICKERS.

### The General and Medical Work of a Deserving Mission.

It was a happy little family group, one of many gathered outside Victoria Station on the last day of September. Children of all ages were seated on the luggage, while mother walked round and counted the bundles. These were bulky and varied—chairs and bedding, pots and pans, oil-stoves, buckets, and impedimenta of every description. "Yus," said Mrs. Brown, of Tidal Basin, pointing to one of her numerous offspring who was proudly wearing his arm in a sling, " 'e broke 'is arm, o' course. 'E always does something every year in the 'opping. But them there nurses *do* look after yer fine." And with this sentiment the Smiths of Stepney and the Kemps of Canning Town whole-heartedly agree.

They are all from the Boughton district, midway between Faversham and Canterbury, one of the areas worked every year by the Hop-Pickers' Mission under the auspices of the Church of England Temperance Society. Last year the operations of the Mission were more widely extended than has been possible since pre-war days.

The Boughton district alone covered fourteen miles (exclusive of the Harbledown gardens two miles from Canterbury), and was worked by a band of nine missioners, including two women medical students from King's College. In Boughton itself the C.E.T.S. dispensary was worked by a trained nurse, who was generally responsible for the medical work of the area. A branch dispensary and dressing-station was opened at Selling, and here minor ailments and injuries of every description were treated after picking hours were over. The nurses were extremely keen on their work, and much impressed the pickers by their assiduous visiting of the various farms during working time, and especially in the rain.

"GETTING READY FOR HOME."

### COMMON AILMENTS.

The daily visiting of both the gardens and the encampments was by no means the least important part of the nurses' work, as it often happened that, when a member of the family (or sometimes the mother herself) was found to be too unwell to accompany the rest to the place of picking, the sufferer was simply left behind in the hut for the day. As several cases of pneumonia occurred (at one distant camp in particular) there is no doubt much useful preventive work was done in this way. Of course, all serious cases were sent to hospital. A record was made of every case treated at the dispensaries—at one alone there were over 1,700 during the month, of which two-thirds were children. Impetigo was common, burns and sores abounded, while on several occasions the staff were called to cases of severe burns caused by falling bodily into the unprotected camp-fires, too suggestive of bonfire night. It is generally felt that some steps ought to be taken to ensure the safety of small children where this real danger is concerned. An attempt to promote in some degree the welfare of the pickers' babies took the form of the opening of a crèche in one of the districts visited by East Enders from Stepney, Poplar, Whitechapel, and Canning Town. The tinies from three weeks to three years were received here from 6.30 A.M. to 6 P.M., tended, amused, and fed in tents provided by the Mission and set up in a pleasant meadow kindly lent by the owner of the farm.

### HOPES FOR NEXT YEAR.

It is hoped next season to extend both this and the medical work of the Mission, and contributions of toys, clothing, linen (for bandages), or money will be thankfully received at the offices of the Mission at 64 Burgate Street, Canterbury.

In every one of the districts worked by the Mission the greatest kindness and ready assistance has been received from the farmers and their bailiffs, as well as the clergy and doctors of the neighbourhood. And in this connection it may not be amiss to record an incident that occurred one Sunday evening in September last year. One of the nurses was summoned in haste to a woman suddenly taken ill in one of the Harbledown encampments. On arrival she found a miscarriage was impending, and the distracted husband was dispatched to the main road not far from the farm to stop the first passing motor and ask to be driven to Canterbury to fetch the doctor. Soon a big touring-car came along and stopped in obedience to his signal. On hearing his request the occupant announced his readiness to come himself. It was a London doctor who thus so generously interrupted his journey late on a Sunday evening to go to the assistance of an unknown woman in need of his aid. He did all he could in the one-roomed hut of corrugated iron, lit by a guttering candle, and then resumed his journey, the richer by the gratitude of husband and nurse. So impressed was the doctor in another area by the work of the Missioners that on their departure he sent them a written commendation.

But it is from the people themselves that one hears the things that warm the workers' hearts on days that are wet or weary. For the Mission to Hop-Pickers is real hard work under circumstances that are not always either clean or comfortable. But the nurses love it, and in many cases come back to it year after year. But there is still scope for increased effort, for more nurses, wider areas, many more dispensaries. Who will help on the good work? The Secretary to the Mission is the Rev. C. F. Tonks, 64 Burgate Street, Canterbury, to whom all letters should be addressed.

## Figures and Tables

**Figure f1:**